# Splenectomy for Solitary Splenic Metastasis in Recurrent Papillary Thyroid Cancer. A Case Report and Literature Review

**DOI:** 10.1155/2020/2084847

**Published:** 2020-05-04

**Authors:** Antonio Maffuz-Aziz, Gabriel Garnica, Silvia López-Hernández, Janet Pineda-Diaz, Javier Baquera-Heredia, Patricia López-Jiménez

**Affiliations:** ^1^Department of Surgical Oncology, American British Cowdray Medical Center, Mexico City, Mexico; ^2^Department of Surgical and Molecular Pathology, American British Cowdray Medical Center, Mexico City, Mexico

## Abstract

Thyroid cancer is the most common endocrine malignancy, presenting with 23 500 new cases per year in the United States. About 7-23% of the patients will present recurrent metastases disease during follow-up. The classic variant of papillary carcinoma is less aggressive compared to its other variants like diffuse sclerosing, tall cell or columnar cell, and insular variants, and the sites to which this metastasizes is already well identified. Metastasis to the spleen is an extremely rare manifestation of papillary thyroid cancer. To date, only 3 cases have been reported in the literature. Herein, we present a 52-year-old male, who developed spleen metastases, 2.4 years after total thyroidectomy and central neck dissection followed by radioactive iodine ablation and seven months after treatment with sorafenib for lung metastases. The splenic lesion was detected in surveillance studies. This case highlights that splenic metastasis, although rare, may occur even in a patient with a locoregional and systemic controlled thyroid cancer and that it can be treated safely with surgical resection.

## 1. Introduction

Thyroid cancer is the leading cause of endocrine cancer and represents 2.1% of all cancer cases worldwide. About 90% are well-differentiated thyroid carcinoma (DTC); papillary cancer is the most common histology [1]. The presence of distant metastases at the time of diagnosis is 4%, and 7-23% during follow-up; in nearly 53% of cases, the relapse is reported in locoregional cancer, 28% in local relapse, and 13% distance metastasis is present; of these, 6% of cases have mixed relapses [2]. It has been reported that a global survival at 10 years is in a range of 25–70% [3].

The most common distant metastasis sites are the lungs and bone with 69.0% and 7.1%, respectively [4]. Rare metastasis site locations are extremely low; they have been identified in sites such as the liver, adrenal gland, central nervous system, kidney, and skin and have been reported in 1.85% of cases [5].

The presentation of spleen metastases of a primary thyroid cancer is even more rare; to date, only 3 cases have been reported [6–8]. In this article, we present a case of splenic recurrence of papillary thyroid cancer and a literature review.

## 2. Case Presentation

A 52-year-old male presented with dysphonia of 2 months; laryngoscopy was performed identifying right vocal cord paralysis; extension studies identified a tumour dependent on the right thyroid lobe, with oesophageal infiltration and tracheal displacement, with no evidence of cervical lymph nodes. Total thyroidectomy with partial resection of the oesophagus and lymphadenectomy of the central compartment was performed. The pathology report was classic papillary thyroid carcinoma 3.6 cm in tumour size, and mixed pattern, with extra thyroid extension, and 1/7 lymph nodes with metastases. Postoperative iodine-131 dose of 200 mCi was delivered, with subsequent iodine-131 tracing that reported small remnant of functional thyroid tissue in the thyroid bed. He continued hormone replacement therapy and surveillance.

At 16 months of surveillance, right basal pulmonary nodule was identified in X-ray; then, whole-body iodine-131 scan and thyroglobulin levels were negative, so 18F-fluorodeoxyglucose-positron emission tomography (18F-FDG PET/CT) was performed which was positive for bilateral pulmonary tumour activity; this being the only place where distant metastasis was found at the time of the study, the spleen was normal. Thoracoscopy was performed, where pulmonary metastases were confirmed secondary to well-differentiated papillary thyroid cancer. Treatment with sorafenib was started, assessing complete pulmonary control after 12 months of treatment.

**Figure 1:**
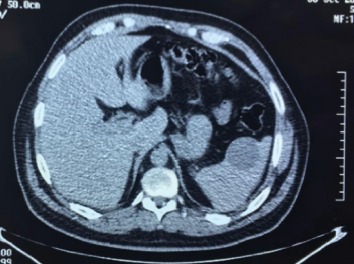
Abdominal CT showing cystic lesion in the spleen of 40 mm diameter.

**Figure 2:**
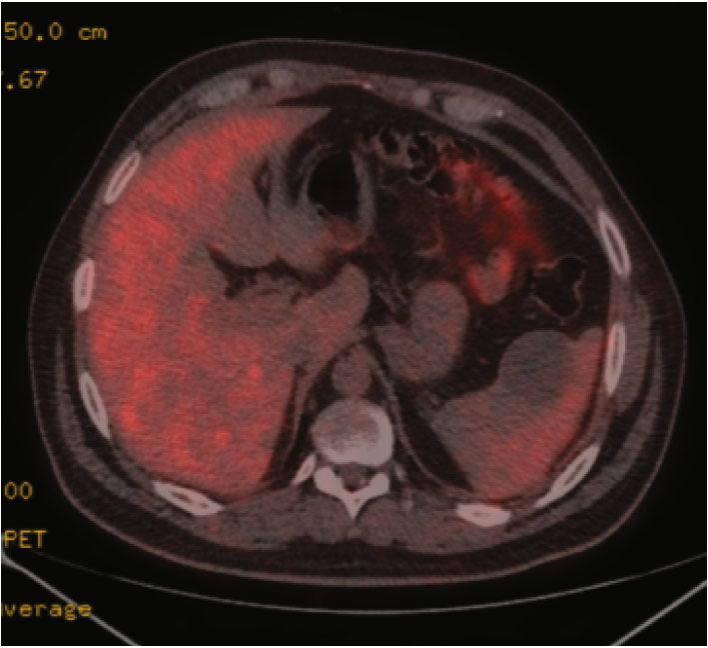
PET/CT images show a 40 mm lesion without increased metabolic activity.

Seven months after finished sorafenib treatment, and 29 months from initial treatment, 18F-FDG PET/CT was performed, in which there was no evidence of metabolic activity in the lung or in any other organ; however a cystic lesion was found in the spleen 10 mm in diameter without metabolic activity. A control 18F-FDG PET/CT at 6 months showed the lung without evidence of disease, and the splenic lesion grew to a diameter greater than 40 mm; it was observed without metabolic activity (Figure 1). Due to the increment of size and risk of spontaneous rupture, it was decided to perform splenectomy (Figure 2).

**Figure 3:**
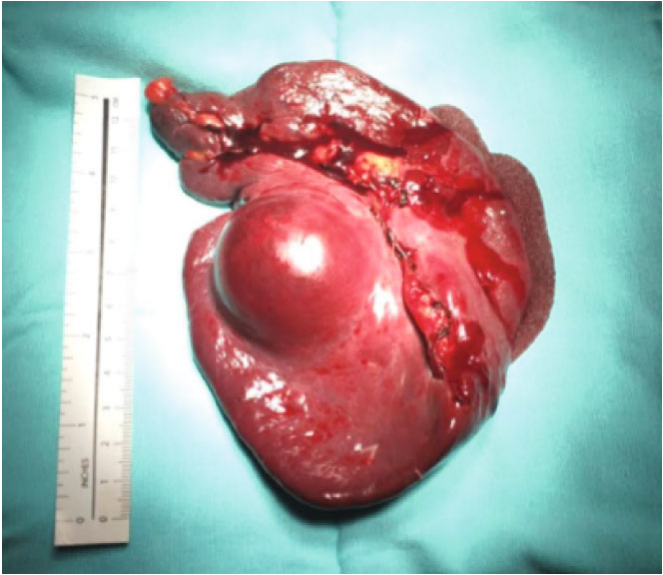
Pathology macroscopic picture shows the spleen with a cystic lesion of 5.5 cm.

**Figure 4:**
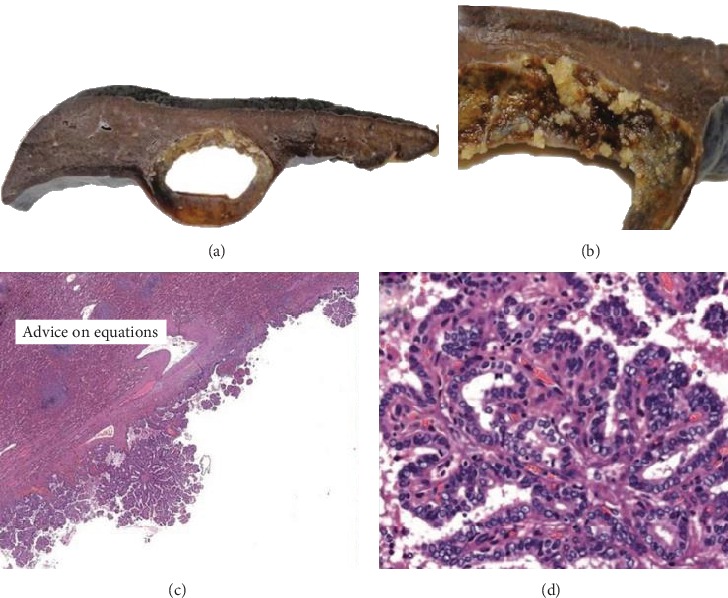
(a) Ovoid splenic lesion of cystic appearance, 5.5 cm major axis on the wall. (b) Papillary projections are observed. (c) Photomicrograph in which simple papillae protruding from the cyst wall (haematoxylin and eosin, 4x) are observed. (d). At a higher magnification, cuboidal cells with clear nuclei and bars, characteristic of papillary thyroid carcinoma (haematoxylin and eosin, 20x) are observed.

**Figure 5:**
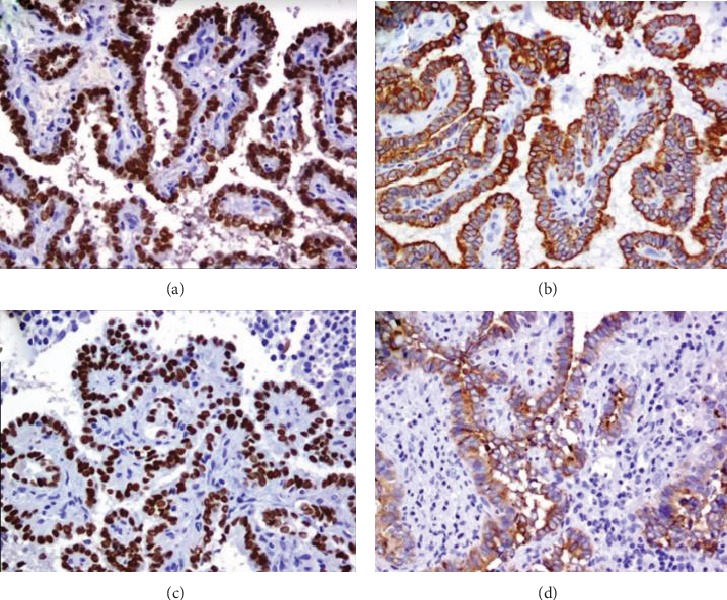
Photomicrographs of the immunohistochemical study performed on the splenic lesion. (a) PAX8 diffuse nuclear positive. (b) Diffuse cytoplasmic positive thyroglobulin. (c) TTF-1 diffuse nuclear positive. (d) CK19 diffuse cytoplasmic positive.

Pathology reported a 5.5 × 5.5 × 4 cm semi ovoid tumour, cystic-looking lesion, with serous content, wall cut 0.2 cm, with simple papillary formations (Figure 3). In sections with haematoxylin and eosin staining, the neoplastic proliferation cyst is with a papillary growth pattern, and cuboidal cells, nuclear pleomorphism, empty nuclei and abundant nuclear bars are without mitosis (Figure 4). In immunohistochemistry, diffuse cytoplasmic positive thyroglobulin, CK19 diffuse cytoplasmic positive, TTF-1 diffuse nuclear positive, PAX8 diffuse nuclear positive, and CK5/6 negative were observed (Figure 5). The diagnosis was papillary thyroid carcinoma with a cystic pattern, of conventional type, well differentiated, with focal microcalcification and intraluminal xanthomatous response, without extracapsular extension. No areas of tall, columnar, or oncocytic cells were identified. There were no poorly differentiated or anaplastic areas.

## 3. Discussion

The presence of spleen metastases from solid tumours is extremely rare and generally exists in the context of a multiorgan disease. The presence of isolated spleen metastases has been reported <1% in autopsy studies; however, it is associated in 17 to 61% with metastases in other distant organs [9]. In the present case, spleen metastases present after treating lung metastases. Although the frequency of metastatic lesions of solid organs to the spleen is rare (2.3-7.1%), it is the most common sites of origin in breast (22.9%), lung (20.2%), colorectal (9.4%), ovary (9%), and stomach (6.9%) cancer [10]. The reason why this type of dissemination is rare is still poorly understood; lack of afferent lymphatic vessel, the splenic capsule, the immunological capacity of the spleen parenchyma cells (macrophages and lymphocytes), and the angled and spiral shape of the splenic artery constitute barrier methods for the presence of metastases in this organ [11].

To date, only 3 cases of thyroid metastases to the spleen have been reported. The first case was reported by Paolini et al. [7]; a patient with history of follicular thyroid cancer, which developed lung and spleen metastases; the patient was diagnosed with splenomegaly and infiltration to the diaphragm, colon, pancreas, and stomach. The second case reported was by Mayayo et al. [6]; with poorly differentiated thyroid carcinoma, the patient presented abdominal pain at 6 months of surveillance. Spleen, liver, and pancreas metastases were identified. The diagnosis was made by fine-needle aspiration cytology (FNA). And the last case reported by Kand et al. [8] was in a 50-year-old patient with a follicular variant of a papillary carcinoma, who was diagnosed with an iodine-131 uptake study, which was captured at a diffuse level throughout the spleen, in addition to associating bone lesions. The definitive diagnosis was made using FNA as well.

Our patient was diagnosed incidentally in surveillance studies; he had no symptoms of abdominal pain and it seemed only a cystic lesion.

Before 1990, when imaging techniques were not used effectively, splenic metastasis rates were between 2.3% and 7.1% and most of them were found during autopsies or were just encountered coincidentally [10], because they are mostly asymptomatic. Therefore, studies such as 18F-FDG PET/CT currently have an important tool for detection. In a study performed on 68 oncology patients with FDG avid malignancy and solid splenic masses on anatomical imaging, 18F-FDG PET/CT had 100% accuracy in characterizing lesions as benign or malignant. The sensitivity, specificity, positive predictive value, and negative predictive value of 18F-FDG PET/CT in differentiating benign from malignant solid splenic lesions in patients with and without malignant disease are 100%, 100%, 100%, and 100% versus 100%, 83%, 80%, and 100%, respectively. It should however be kept in mind that non-FDG-avid tumours, such as some renal or thyroid cancers, may metastasize to the spleen [12, 13].

Although the information in the literature regarding the relationship between 18F-FDG PET/CT and the diagnosis of metastatic spleen lesions is only for solid tumours, the probable explanation is that most well-differentiated thyroid carcinomas are relatively slow growing and can be 18F-fluorodeoxyglucose negative [14]. Several studies have reported that it has a high sensitivity (up to 85%) and specificity (up to 95%) for distant metastases in patients with well-differentiated thyroid cancer [15].

Use of FNA is a useful diagnosis tool, since a sensitivity of 98.4%, a positive predictive value of 99.2%, and 98.1% accuracy for diagnosis and < 1% of complications have been reported [16], although it is generally avoided because of the risk of intra-abdominal bleeding or dissemination in some cases.

For this reason and based on the clinical evolution that the patient had, which was presented as a growth of the lesion, we decided to perform splenectomy because it was a unique and viable cystic lesion for resection.

Pathologic findings, the presence of isolated epithelial cells or forming three-dimensional groups with round nuclei, with inclusions or bars, are characteristics that should be suspected in a thyroid origin, especially in patients with a history of papillary thyroid carcinoma [6].

The long-term survival after splenectomy in patients with metachronous splenic metastasis from thyroid papillary cancer is unknown because of the limited number of reported cases in the literature; however, based on the data obtained from the study by Madani et al. [17] where they analysed a study of 492 patients with thyroid cancer and rare sites of metastasis, they mention patients with generally aggressive tumours, with a global survival of 60 months and disease-free period of 84 months. Other sites like colorectal carcinoma where the 1-year survival rate was 86.6%, and median survival time is 66.6 months [18], In metastases secondary to melanoma, median overall survival after splenectomy is 11 months, with a survival of 23 months for the subgroup of patients treated for a solitary lesion [19].

Distant metastasis is considered an important prognostic factor in papillary thyroid cancer, which affects survival. The 5-year survival rate is almost 100% for localized papillary, 99% for locoregional cancer and 78% for metastatic papillary thyroid cancer [20].

For patients with only lung metastases, the survival rate at 10 years is 73.6%, which are significantly higher than patients with multiple organ metastases for whom the 10-year survival rate is 34.3% [21].

## 4. Conclusions

Papillary thyroid cancer is a very common neoplasm; there a lot of information in articles and guides regarding its behaviour and management options. However, on rare behaviour, uncommon site metastases can occur, and its management is not well defined.

## Data Availability

The [DATA TYPE] data used to support the findings of this study are included within the article.
